# CCL20/CCR6 axis mediates macrophages to promote proliferation and migration of ESCs by blocking autophagic flux in endometriosis

**DOI:** 10.1186/s13287-022-02981-2

**Published:** 2022-07-15

**Authors:** Jiahuan Tan, Tenghan Xu, Yanling Gou, Honglin Wang, Zongwen Liang, Yingying Cao, Han Wang, Yangyang Yu, Na Jiao, Zongfeng Zhang

**Affiliations:** 1 Department of Obstetrics and Gynecology, Second Affiliated Hospital of Harbin Medical University, 148 Baojian Road, Harbin, 150086 China; 2 Department of Obstetrics and Gynecology, Luohe Central Hospital, Luohe, China; 3 Department of Obstetrics and Gynecology, Fourth Affiliated Hospital of Harbin Medical University, Harbin, China

**Keywords:** Endometriosis, Macrophage, CCL20/CCR6 axis, Autophagic flux, TFEB, Lysosomal function

## Abstract

**Background:**

Endometriosis (EMs) is a common benign gynecological disease that affects approximately 10% of females of reproductive age. Endometriosis ectopic lesions could recruit macrophages, which in turn facilitates endometriosis progression. Several studies have indicated that CCL20 derived from macrophages activates the expression of CCR6 in several cells and induces cell proliferation and migration. However, the function of the CCL20/CCR6 axis in the interactions between macrophages and endometriotic stromal cells (ESCs) in EMs has yet to be elucidated.

**Methods:**

Ectopic and normal endometrial tissues were collected from 35 ovarian endometriosis patients and 21 control participants for immunohistochemical staining. It was confirmed that macrophages secreted CCL20 to promote CCR6 activation of ESCs during co-culture by ELISA, qRT-PCR and western blot analysis. CCK8 and Edu assays were used to detect cell proliferation, and wound healing and Transwell assay were used to detect cell migration. Autophagic flux was detected by measuring the protein expression levels of LC3 and P62by western blot and analyzing the red/yellow puncta after ESCs were transfected with mRFP-GFP-LC3 double fluorescence adenovirus (Ad‐LC3). Lysosomal function was tested by quantifying the fluorescent intensities of Lyso-tracker and Gal3 and activity of acid phosphatase. In addition, co-IP experiments verified the binding relationship between CCR6 and TFEB. Finally, the suppressive effect of CCL20-NAb on endometriosis lesions in vivo was demonstrated in mice models.

**Results:**

We demonstrated that macrophages secreted CCL20 to promote CCR6 activation of ESCs during co-culture, which further induced the proliferation and migration of ESCs. We observed that the CCL20/CCR6 axis impaired lysosomal function and then blocked the autolysosome degradation process of autophagic flux in ESCs. The combination of CCR6 and TFEB to inhibit TFEB nuclear translocation mediates the role of the CCL20/CCR6 axis in the above process. We also found that co-culture with ESCs upregulated the production and secretion of CCL20 by macrophages. The suppression effect of CCL20-NAb on endometriosis lesions in vivo was demonstrated in mice models.

**Conclusions:**

Our data indicate that macrophages block TFEB-mediated autolysosome degradation process of autophagic flux in ESCs via the CCL20/CCR6 axis, thereby promoting ESC proliferation and migration.

**Supplementary Information:**

The online version contains supplementary material available at 10.1186/s13287-022-02981-2.

## Background

Endometriosis (EMs) is a common benign gynecological disease defined as the presence of endometrium-like tissue outside the uterus [1], and it affects approximately 10% of females of reproductive age. The true incidence of EMs in the general population is uncertain because the diagnostic challenge in EMs is multifaceted due to the lack of specific symptoms, sensitive biomarkers and awareness on the part of the public and practitioners. EMs clinically presents several hallmarks yet nonspecific symptoms such as dysmenorrhea, dyspareunia, chronic pelvic pain and infertility, as well as induced depression and anxiety [2]. Thus, in-depth investigations of the pathogenesis of EMs are urgently required to explore novel therapeutic targets. To date, there are multiple theories to explain the pathogenesis of endometriosis, including retrograde menstruation theory, coelomic metaplasia theory and stem cell theory. Among them, the retrograde menstruation theory proposed by Sampson is broadly accepted. Since it cannot answer why some females with the dissemination of endometrial cells out of the uterine cavity develop endometriosis and not others, it is suggested that there are several factors involved in the process of lesion adhesion, invasion and angiogenesis, including aberrant hormone levels, genetic susceptibility, immune dysfunction and others [3–6]. Numerous studies have demonstrated that dysfunction of the immune microenvironment is a pathophysiological mechanism of EMs, including aberrant immune cell contents and cytotoxicity [7–9]. Neutrophils and macrophages are cells to be firstly recruited in response to the advent of endometrial debris in the peritoneal cavity. It has been reported that the number of neutrophils is augmented in peritoneal fluid among women with endometriosis compared with healthy female controls [10]. Macrophages are the largest population of immune cells in peritoneal fluid among endometriosis patients, and they are present at higher concentrations and proportions among endometriosis patients than among controls [10, 11]. Furthermore, aberrant alterations in the local microenvironment have been shown to induce the polarization of macrophages toward M2-phenotype, which is beneficial to the growth and angiogenesis of lesions [12]. Other altered immune cell populations, such as function-deficient NK cells [13, 14] and a reduced number of mature dendritic cells [15], lead to ectopic endometrial fragments evading immune surveillance in the peritoneal cavity and promote undesirable biological behaviors such as the proliferation and migration of lesions.

Our group has been devoted to studying macrophages for a long time, and our previous study demonstrated that macrophages are recruited to EMs lesions and promote the development of disease [16]. However, the detailed mechanism by which recruited macrophages contribute to the progression of EMs remains unclear. Therefore, this study attempts to explore the role of macrophages in endometriosis. Macrophages mainly function as major secretory cells and release a diverse repertoire of chemokines and cytokines which are crucial mediators in modulating the immune response. CC chemokine ligand 20 (CCL20), also called macrophage inflammatory protein-3α (MIP-3α), specifically binds to chemokine (C–C motif) receptor 6 (CCR6). The CCL20/CCR6 axis plays a crucial role in the development of cancers and autoimmune diseases by promoting cell proliferation and migration and remodeling the immune microenvironment [17, 18]. Previous studies have indicated that CCL20 secreted by tumor-associated macrophages (TAMs) activates the expression of CCR6 in tumor cells and then induces cell proliferation and migration, as well as epithelial–mesenchymal transition (EMT) [19–21]. Although endometriosis is a benign gynecological disease, it has several biological behaviors and immunosuppressive microenvironment similar to those of tumors to promote disease progression. Since the mechanism by which macrophage-involved immune dysfunction contributes to endometriosis progression is imperfect, investigations of the CCL20/CCR6 axis in endometriosis are of interest. Recently, some studies have explored the role of the CCL20/CCR6 axis in immunological interactions in endometriosis and examined its existence in ectopic tissues [22–24]. Dienogest inhibits IL-1β stimulation-induced CCL20 expression in endometriotic epithelial cells, which is mediated by progesterone receptor B [25]. Stimulation by IL-1β, TNF-α and IL-17A induces increased secretion of CCL20 from ESCs, which ameliorates the recruitment of CCR6 + TH17 cells to ectopic lesions [26]. Elevated CCL20 concentrations in the peritoneal fluid from endometriosis patients also recruit more Treg cells, leading to an increased proportion of Treg cells in the peritoneal fluid, thereby promoting the progression of endometriosis [27].

Autophagy is a novel type of programmed cell death that is organized into three main forms: macroautophagy, microautophagy and chaperone-mediated autophagy [28]. The most-studied type of autophagy is macroautophagy, a collection of lysosomal degradation processes contributing to intracellular homeostasis [29], and the term “autophagy” refers to macroautophagy. Autophagy is responsible for numerous pathophysiological processes, including cellular metabolism, inflammation, tumorigenesis and immune escape [30–32]. Currently, the effect of autophagy on the initiation and progression of EMs is inconclusive. Several studies report that autophagy promotes the initiation and progression of EMs [33, 34], while others document the inhibitory effect of autophagy on EMs [35–37]. Many lines of evidence indicate that autophagy is associated with the CCL20/CCR6 axis, which supports the next step in our study [38–45]. Hence, a comprehensive understanding of EMs pathogenesis necessitates insight into the interaction between immune regulation and autophagy.

In order to investigate the role of the CCL20/CCR6 axis in macrophages and ESCs in EMs, we detected the effect of inhibiting the CCL20/CCR6 axis in co-culture system on the progression of EMs. We screened the signaling pathways whose states were altered by the macrophage-mediated CCL20/CCR6 axis and selected the obviously altered autophagy for further study. We found that the CCL20/CCR6 axis blocked the TFEB-mediated autolysosome degradation process of autophagic flux in ESCs, thereby promoting ESC proliferation and migration. These findings suggest that the CCL20/CCR6 axis is required for macrophages to regulate the proliferation and migration of ESCs in EMs. Therefore, targeted inhibition of the CCL20/CCR6 axis eliminates macrophage-mediated pro-proliferation and migration of ESCs, which is an effective strategy to inhibit EMs progression.

## Materials and methods

### Patients and tissue samples

A total of 35 female patients with EMs undergoing laparoscopic surgery and who were diagnosed by pathological examination were enrolled at the Department of Gynecology, the Second Affiliated Hospital of Harbin Medical University, from June 2019 to June 2021. Twenty-one women without EMs or adenomyosis undergoing operative surgery for other benign gynecological diseases, such as uterine leiomyomata, were considered as the control group. (Detailed information on these patients is presented in Table 1.) Samples from these participants in the proliferative phase of the menstrual cycle were collected during surgical treatment and then divided into two portions. One portion was used for primary cell culture for a series of in vitro studies and was immediately transported to the laboratory at low temperature in Dulbecco’s modified Eagle medium (DMEM) (Biological Industries, Israel), while the other portion was subjected to paraffin sectioning and fixed in 4% PFA (Beyotime Biotechnology, China). Participants recruited for this study signed written informed consent forms. The study was approved by the Ethical Committee of the Second Affiliated Hospital of Harbin Medical University (approval number: KY2016-040).

**Table 1:** Clinical characteristics of female with and without (control) endometriosis

	Control (*n* = 21)	Endometriosis (*n* = 35)	*P* value
Age (year)	41.43 ± 1.68	38.94 ± 1.19	0.221
BMI (kg/m^2^)	23.37 ± 0.49	23.44 ± 0.56	0.923
CA125 level (U/mL)	18.98 ± 2.46	83.35 ± 20.09	0.016
Menstrual average cycle (day)	28.25 ± 0.65	29.83 ± 1.18	0.279
Menstrual duration (day)	5.04 ± 0.28	5.74 ± 0.26	0.076
r-AFS stage			
Stage I (minimal)		0/35	
Stage II (mild)		7/35	
Stage III (moderate)		19/35	
Stage IV (severe)		9/35	

Statistical analysis was performed using Student’s *t* test. Data are mean ± SD. AFS, American Fertility Society

### Cells and cell culture

Primary ectopic endometrial stromal cells (ESCs) from 35 patients with ovarian endometriosis were isolated and cultured, as described previously [16]. Ectopic endometrial tissues were cut into pieces of approximately 1 mm × 1 mm size and digested with 4% type IV collagenase (Sigma, USA) at 37 °C in a water-bath pot for 40–60 min. The mixed cell suspension was filtered to remove debris and epithelial cells and then washed three times to remove erythrocytes. The ESC pellets were resuspended in DMEM with 15% fetal bovine serum (FBS) (Biological Industries, Israel) and 1% penicillin–streptomycin (Gibco, USA), plated on a cell culture dish and cultured in an incubator (5% CO_2_, 37 °C). After cell attachment, the original medium was replaced. ESCs were passaged with 0.25% trypsin–EDTA (Gibco, USA) for digestion at approximately 80% confluence. The ESCs identified by immunofluorescent staining for vimentin (+) and cytokeratin 7 (−) (Additional file 1: Fig. S1) were used for subsequent in vitro experiments in triplicate.

THP-1 cells (acute monocytic leukemia cell line) purchased from ScienCell were suspension-cultured in Roswell Park Memorial Institute 1640 (RPMI-1640) (Biological Industries, Israel) medium with 10% FBS and 1% penicillin–streptomycin, and passaged regularly. After incubation with 100 ng/ml phorbol 12-myristate 13-acetate (PMA) (Sigma, USA) for 48 h, THP-1 monocytes were efficiently differentiated into macrophages. Differentiated macrophages adhered to the dishes, and the cell morphology was altered.

The 0.4 μm-pore polycarbonate Transwell inserts (Corning, USA) were used in the co-culture system. ESCs were co-cultured with differentiated macrophages at a ratio of 1:1.

### Immunohistochemical staining

Immunohistochemical staining was performed by standard methods. After antigen retrieval and blocking, paraffin sections were sequentially incubated with the corresponding primary antibodies (dilution ratios are shown in Table 2) overnight at 4 °C and with the secondary antibody for 1 h at room temperature. A diaminobenzidine kit (ZSGB-BIO, China) was applied for chromogenic detection, followed by counterstaining with hematoxylin. Finally, paraffin sections were photographed by light microscopy and the number of positive cells was counted. Negative controls were conducted without primary antibodies.

**Table 2:** Details of antibody used in experiments

Application	Antibody	Catalog number	Dilution	Source
IHC				
	CD68	25747-1-AP	1:2000	Proteintech
IF				
	Vimentin	10366-1-AP	1:50	Proteintech
	Cytokeratin 7	15539-1-AP	1:50	Proteintech
	CCR6	130120598	1:50	Miltenyi Biotec
	Gal3	sc-32790	1:50	Santa Cruz
	LC3	14600-1-AP	1:100	Proteintech
	LAMP2	66301-1-Ig	1:50	Proteintech
	TFEB	13372-1-AP	1:50	Proteintech
WB				
	CCR6	DF10207	1:1000	Affinity
	GAPDH	60004-1-Ig	1:20000	Proteintech
	P62	18420-1-Ap	1:1000	Proteintech
	LC3	14600-1-AP	1:2000	Proteintech
	β-actin	66009-1-Ig	1:10000	Proteintech
	TFEB	13372-1-AP	1:500	Proteintech
	Histone H3	17168-1-AP	1:1000	Proteintech
	Ki67	AF0198	1:1000	Affinity

IHC, Immunohistochemical; IF, Immunofluorescence; and WB, Western blot

### Cell proliferation assay

The proliferative capability of ESCs was assessed by CCK8 and Edu assays.

**Fig. 1:**
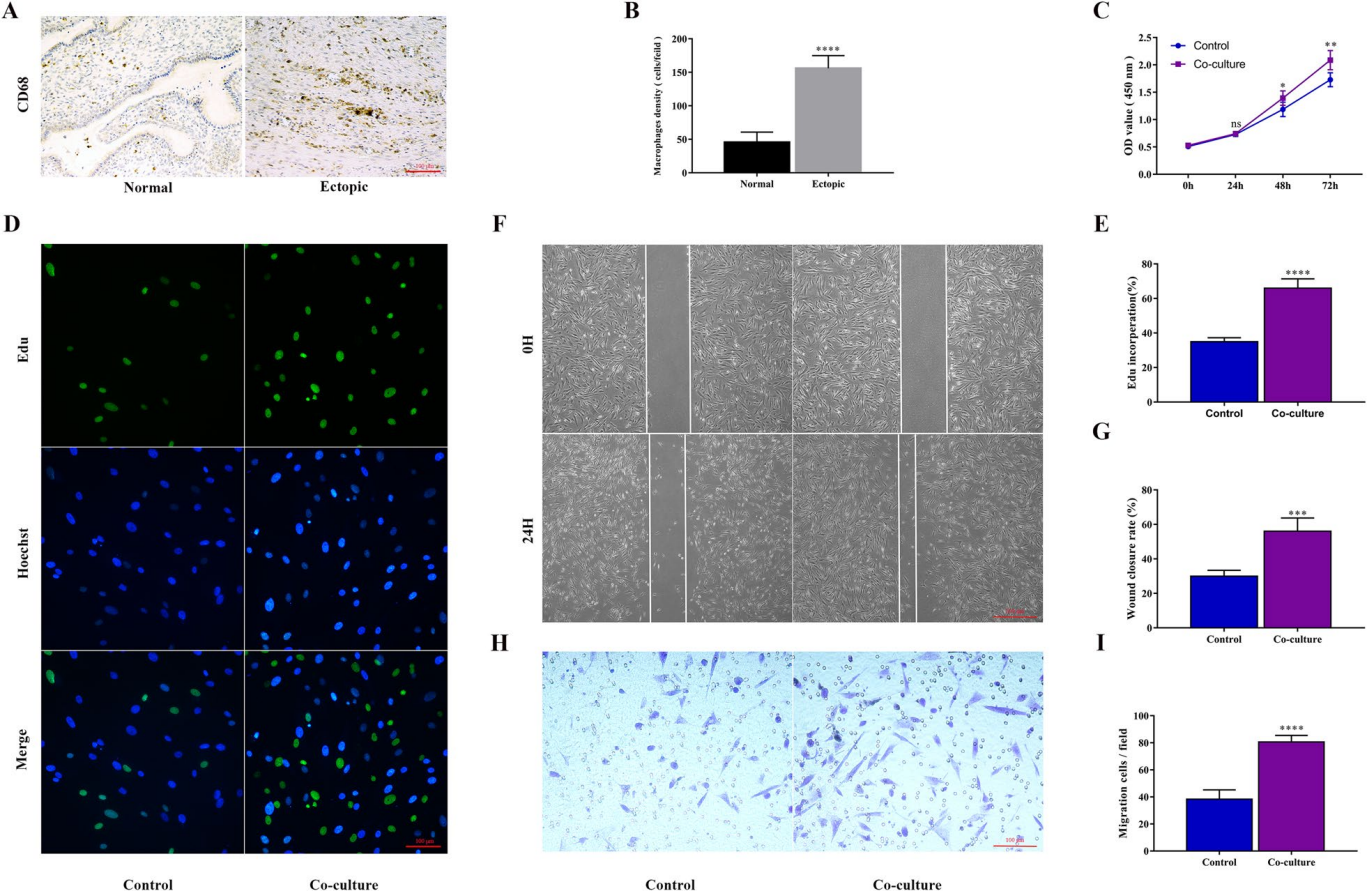
Macrophages promote the proliferation and migration of ESCs in EMs. **A** Representative immunohistochemical detection of macrophages, stained with CD68, in human endometriosis ectopic lesions and normal endometrial tissues. (original magnification 200 ×) **B** Quantification of macrophage density (cells/per field at 200 × magnification) in human endometriosis ectopic lesions (n = 10) and normal endometrial tissues (n = 10). **C** Proliferative capability of ESCs with and without co-culture treatment assessed by CCK8 assay. **D** Proliferative capability of ESCs with and without treatment of co-culture assessed by Edu assay. (original magnification 200 ×) **E** Quantification of Edu incorporation, Edu incorporation rate (%) = Edu positive cells (green)/Hoechst-positive cells (blue). **F** Migration capability of ESCs with and without treatment of co-culture assessed by wound healing assay. (original magnification 40 ×) **G** Quantification of wound closure rate, wound closure rate (%) = (Scratch distance 0 h—Scratch distance 24 h)/Scratch distance 0 h. **H** Migration capability of ESCs with and without treatment of co-culture assessed by Transwell assay. (original magnification 200 ×) **I** Quantification of migrating cells per field at 200 × magnification. Data are presented as the mean ± SD of n = 3 independent experiments. Statistical analysis was performed using Student’s *t* test (**B**) and Mann–Whitney test (**C**, **E**, **G**, **I**). ****p < 0.0001, ***p < 0.001, **p < 0.01, *p < 0.05, ns, non-significant vs. control group

ESCs were seeded in 96-well plates (1 × 10^4^/mL) and divided into several groups based on different treatments after attachment, with five parallel wells for each group. For the experiments shown in Fig. 1, ESCs were co-cultured with macrophages by using the 0.4 μm-pore Transwell inserts or adding macrophage culture supernatants for 48 h. 20 ng/ml recombinant human CCL20 (rCCL20) (MedChemExpress, USA) was added to ESCs culture supernatants, and 2 ug/ml CCL20 neutralizing antibody (CCL20-NAb) (R&D Systems, USA) and 6 nM CCR6 inhibitor (MedChemExpress, USA) were, respectively, added to the co-culture system. For the experiments shown in Fig. 3, TFEB overexpression plasmids were transfected into ESCs incubated with rCCL20, with or without 5 mM 3-Methyladenine (3-MA) (MedChemExpress, USA). 10 μL CCK8 (Dojindo, Japan) was injected and incubated for 2 h after 0 h, 24 h, 48 h and 72 h of treatment. Absorbance at 450 nm was detected by a microplate reader, and then, proliferation curves were plotted according to the OD value.

ESCs were seeded in 24-well plates (2 × 10^4^/mL) and were divided into several groups based on different treatments after attachment, with three parallel wells for each group. Details of these treatments were as described in the CCK8 assay. After treatment for 48 h, an Edu assay was conducted using an Edu cell proliferation kit with Alexa Fluor 488 (Beyotime, China) according to the instructions. After incubation with Edu working reagent at 37 °C for 4 h, ESCs were routinely fixed, washed and permeabilized. Then, ESCs were incubated with Click Additive Solution and nuclear dye Hoechst 33,342, protected from light. The following formula was applied to calculate the proliferative capability of ESCs: Edu incorporation rate (%) = Edu positive cells (green)/Hoechst-positive cells (blue). Experiments were conducted in triplicate.

### Cell migration assay

The migration capability of ESCs was assessed by wound healing and Transwell assays.

ESCs were seeded into six-well plates at 2 × 10^5^/well. After ESCs were cultured to a confluent monolayer, vertical scratches were created with 200 μL pipet tips in the wells, and scraped ESCs were washed with sterile phosphate-buffered saline (PBS) (VivaCell, China). FBS-free DMEM medium was added to wells, and ESCs were treated similarly with that in cell proliferation assay. The scratch distances were photographed and measured at 0 h and 24 h. Wound closure rate (%) = (Scratch distance 0 h—Scratch distance 24 h)/Scratch distance 0 h.

Washed ESCs (3 × 10^5^/mL), resuspended in DMEM without FBS, were inoculated into the 8 μm-pore Transwell upper chamber (Corning, USA), while DMEM with 15% FBS was added into the lower chamber. After the same treatments as the cell proliferation assay for 24 h, the chambers were washed, fixed and stained. Then, chamber membranes were photographed by light microscopy, and the number of migratory ESCs on the lower surface was counted to assess migration capability.

### Enzyme-linked immunosorbent assay (ELISA)

Cell culture supernatant collected from different groups was used to measure CCL20 concentration by ELISA kit (Proteintech, USA). For the experiments shown in Figs. 1 and 5, concentrations of CCL20 in ESC culture supernatant with or without co-culture treatment with macrophages and in macrophage culture supernatant with or without co-culture treatment with ESCs were detected, respectively. After all steps of ELISA, including adding samples, incubating, washing and coloring, were completed according to the manufacturer’s instruction, the absorbance at 450 nm was detected within 15 min. The concentration of CCL20 was calculated based on a standard curve.

### Quantitative real-time PCR (qRT-PCR)

The quality of total RNA was confirmed by Nanodrop 2000, followed by extraction from treated cells using TRIzol (Invitrogen, USA). Total RNA (500 ng) was reverse transcribed with a One-Step gDNA Removal and cDNA Synthesis SuperMix kit (TransGen Biotech, China) to synthesize cDNA (20 μL), and then, qRT-PCR was conducted to quantify gene expression using a Top Green qPCR SuperMix kit (TransGen Biotech, China). The primer sequences for each gene were designed and validated for specificity. The primer sequences used in qRT-PCR were as follows: CCL20-F (5′-GGAATGGAATTGGACATAGCC-3′) and CCL20-R (5′-CCTCCATGATGTGCAAGTGA-3′); CCR6-F (5′-AATCGCTTGAACCCAGAAGC-3′) and CCR6-R (5′-GAGTCTCGCTTTGTCACC-3′); TFEB-F (5′-TGTTGCTGCATGCGCTC-3′) and TFEB-R (5′-CGGCAGTGCCTGGTACAT-3′); TNF-α-F (5′-CCTCTCTCTAATCAGCCCTCTG-3′) and TNF-α-R (5′- GAGGACCTGGGAGTAGATGAG-3′); IL-6-F (5′-TACATCCTCGACGGCATCTC-3′) and IL-6-R (5′-TTTCAGCCATCTTTGGAAGG-3′); GAPDH-F (5′-GGAGCGAGATCCCTCCAAAAT-3′) and GAPDH-R (5′-GGCTGTTGTCATACTTCTCATGG-3′); and β-actin-F (5′-GCTCCTCCTGAGCGCAAG) and β-actin-R (5′-CATCTGCTGGAAGGTGGACA-3′). The relative expression of genes of interest was calculated and normalized to that of GAPDH or β-actin using the 2-ΔΔCt method.

### Transfection

ESCs were seeded at 1 × 10^5^ cells/well into six-well plates and were transfected, respectively, with si-CCR6 (Ribobio, China) and TFEB-overexpressing plasmids (Ribobio, China) at approximately 70% confluence. DNA (2.5 μg) diluted with 125 μL Opti-MEM (Gibco, USA) and 5 μL Lipo3000 (Invitrogen, USA) diluted with 125 μL Opti-MEM were mixed and incubated for 20 min at room temperature. The mixture was added to six-well plates, the supernatants were replaced 8 h later, and transfected ESCs were collected for related Experiments 48 h after transfection. Analysis of transfection efficiency is shown in Additional file 2: Fig. S2 A, B.

Similarly, mRFP-GFP-LC3 double fluorescence adenovirus (Ad‐LC3) (Hanbio, China) was directly transfected (MOI = 30, 50, 100, 300, 500) into ESCs at 80% confluence (Additional file 2: Fig. S2 C). At MOI = 300, the transfection efficiency of Ad-LC3 in ESCs is reliable and its cytotoxicity is low. Transfected ESCs were photographed using confocal microscopy 48 h after transfection. ESCs with different treatments were transfected with Ad‐LC3 in the presence or absence of 100 nM Baf-A1 (MedChemExpress, USA) treatment to suppress the fusion of autolysosomes. The different colors of merged puncta indicated corresponding stages because GFP was quenched rapidly in acidic lysosomes, while mRFP was stable. Thus, yellow puncta represent autophagosomes (mRFP^+^/GFP^+^ fluorescence), while red puncta represent autolysosomes (mRFP^+^/GFP^−^ fluorescence).

### Western blot

Total protein from cells was extracted with RIPA lysis buffer (Beyotime, China) containing phenylmethanesulfonyl fluoride (PMSF) (Solarbio, China) and cocktail (MedChemExpress, USA), and cytosolic and nuclear proteins were separated by a Nuclear and Cytoplasmic Protein Extraction Kit (Beyotime, China). An Enhanced BCA Protein Assay Kit (Beyotime, China) was used to measure the protein concentration of each sample. Protein samples (20 μg/lane) were loaded in SDS-PAGE gels, followed by electrophoresis, and then transfer to 0.22 μm PVDF membrane (Millipore, USA). After blocking, the membranes were sequentially incubated with the corresponding primary antibodies (Table 2) overnight at 4 °C, and secondary antibodies for 1.5 h at room temperature. The membranes were exposed and developed after immersion in ECL reagent (Epizyme, China). The relative target protein levels were equal to the ratios of their gray value to GAPDH or β-actin, which served as internal references.

### Immunofluorescence (IF)

After treatment, ESCs were fixed, perforated and blocked. ESCs were sequentially incubated with the corresponding primary antibodies (dilution ratios are shown in Table 2) overnight at 4 °C, and with fluorescent secondary antibodies and DAPI, respectively, for 1 h and 15 min at room temperature, respectively, protected from light. ESCs were photographed by fluorescence microscopy after sealed with an anti-fluorescence quencher.

### Lyso-Tracker Green staining

Lyso-Tracker Green (Beyotime, China) was diluted with DMEM medium at 1:14,000 to prepare working reagent, and it was preincubated at 37 °C before use. ESCs then were incubated with Lyso-Tracker Green working reagent for 30 min at 37 °C. The working reagent was replaced by fresh medium, and ESCs were observed sequentially by fluorescence microscopy.

### Co-immunoprecipitation (Co-IP)

Cell protein was extracted with lysis buffer for IP (Beyotime, China) containing PMSF and cocktail. After centrifugation, the supernatant was collected, and corresponding primary antibodies at appropriate concentrations according to the instructions were added and mixed overnight at 4 °C on a rotating mixer. Then, the protein supernatant was incubated with pretreated magnetic beads for 4 h at 4 °C for conjugation. The bead–antibody–antigen complex was isolated and harvested by a magnetic separator and resuspended in a mixture of loading buffer and lysis buffer for subsequent immunoblotting.

### Animal experiments

C57BL/6 female mice (eight weeks old) were purchased from the animal center of the 2nd Affiliated Hospital of HMU. Minced uterine fragments from one donor mice were injected intraperitoneally into two recipient mice equally to establish a mice model of EMs, as described previously. Eighteen donor mice were used in this experiment, and EMs mice models were successfully established in thirty-six recipient mice. Mice in the treatment group were injected intraperitoneally with 50 μg CCL20-NAb (R&D Systems, USA) every four days from Day 3, while PBS was injected as control. Mice were killed on day 5, 9 and 14, and uterine and ectopic lesions were collected for immunohistochemistry and measurement. There were six mice in each group per time point. The detailed animal experiment process is shown in Fig. 7A.

### Statistical analysis

Each experiment described above was conducted in triplicates and repeated three times for data collection and analysis, unless specifically stated. All data are shown as the mean ± SD and were analyzed with GraphPad Prism 7 (San Diego, USA). Normal distribution data between two groups were compared using Student’s *t* test, and non-normal distribution data were compared using Mann–Whitney test. Comparisons among multiple groups were made using one-way ANOVA followed by post hoc Tukey test. *p* < 0.05 was considered a significant difference.

## Results

### Macrophages facilitate the proliferation and migration capacity of ESCs in EMs

Immunohistochemical staining of paraffin sections of human tissues showed that the infiltration of macrophages in endometriosis ectopic lesions was significantly higher than that in normal endometrial tissues (Fig. 1A, B), consistent with our previous report [16]. To further explore the function of macrophages in the progression of endometriosis, we measured the proliferation and migration capacity of primary endometriotic stromal cells (ESCs) with and without co-cultured with macrophages by CCK8 (Fig. 1C) and Edu (Fig. 1D, E) assay, wound healing (Fig. 1F, G) and Transwell migration (Fig. 1H, I) assay, respectively. The proliferation and migration abilities of ESCs co-cultured with PMA-treated macrophages were significantly enhanced compared with those of the control group. These data demonstrated that macrophages facilitated the proliferation and migration of ESCs in vitro.

### Macrophages promote the proliferation and migration of ESCs via the CCL20/CCR6 axis in EMs

**Fig. 2:**
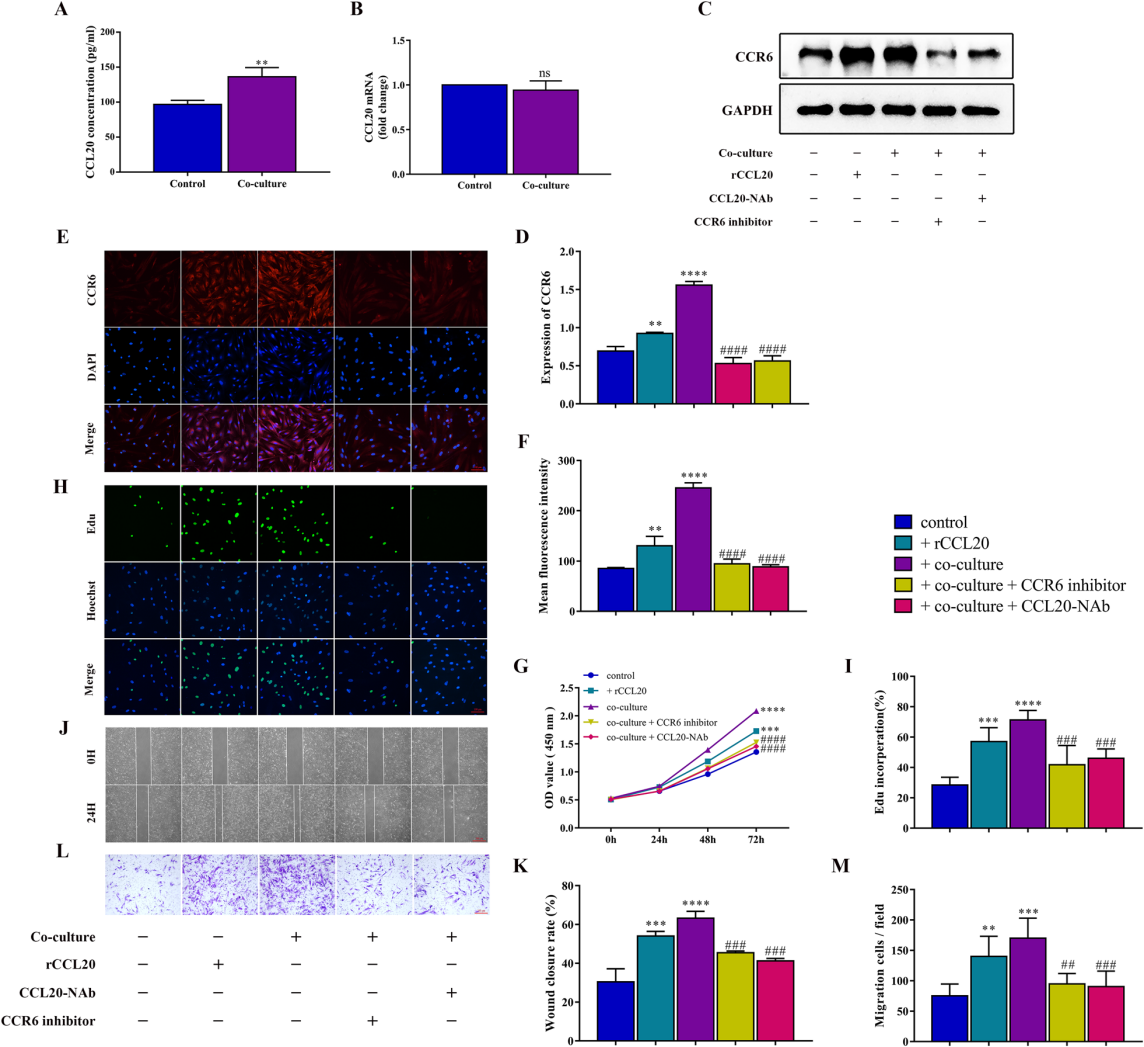
Macrophages promote the proliferation and migration of ESCs via the CCL20/CCR6 axis in EMs. **A** ELISA analysis of CCL20 in ESC culture supernatant with or without co-culture treatment. **B** The mRNA level of CCL20 in ESCs with or without co-culture treatment by qRT-PCR. **C** The protein level of CCR6 in ESCs in different groups by western blot. **D** Quantification of the gray value of western blot bands. **E** Representative immunofluorescence images of CCR6 (red) in ESCs in different groups, Nuclei were stained with DAPI (blue). (original magnification 200 ×) **F** Quantitative fluorescence intensity of CCR6. **G** Proliferative capability of ESCs in different groups assessed by CCK8 assay. **H** Proliferative capability of ESCs in different groups assessed by Edu assay. (original magnification 200 ×) **I** Quantification of Edu incorporation, Edu incorporation rate (%) = Edu positive cells (green)/Hoechst positive cells (blue). **J** Migration capability of ESCs in different groups assessed by wound healing assay. (original magnification 40 ×) **K** Quantification of wound closure rate, wound closure rate (%) = (Scratch distance 0 h—Scratch distance 24 h) / Scratch distance 0 h. **L** Migration capability of ESCs in different groups assessed by Transwell assay. (original magnification 200 ×) **M** Quantification of migrating cells per field at 200 × magnification. Data are presented as the mean ± SD of n = 3 independent experiments. Statistical analysis was performed using Mann–Whitney test (A, B) or one-way ANOVA with Tukey post hoc (D, F, G, I, K, M). ****p < 0.0001, ***p < 0.001, **p < 0.01, ns, non-significant vs. control group. ^####^*p* < 0.0001, ^####^*p* < 0.001, ^##^*p* < 0.01 vs. co-culture group

In the following section, we attempt to explore the mechanism by which macrophages promote the proliferation and migration of ESCs. Macrophages secrete chemokines for effective intercellular signaling interactions to modulate the immune microenvironment in tumors and other diseases, which additionally influence the progression of disease. However, the profile of chemokines derived from macrophages in EMs remains to be adequately elucidated. CCL20, alternatively called macrophage inflammatory protein-3α (MIP-3α), is involved in remodeling the immune microenvironment and promoting disease progression. Numerous studies have indicated that CCL20 derived from macrophages activates the expression of CCR6 in several tumor cells and then induces cell proliferation and migration, as well as EMT. Thus, we intended to explore the role of CCL20 in the interaction between macrophages and ESCs. We analyzed the concentration of CCL20 in the supernatants of ESCs by ELISA and found that the concentration of CCL20 in the co-cultured group was significantly upregulated compared with that in the control group (Fig. 2A). However, there was no difference in mRNA expression of CCL20 in ESCs with different treatments detected by qRT-PCR (Fig. 2B). The results indicated that the upregulated CCL20 in the supernatant of co-cultured ESCs was derived from the secretion of macrophages. CCR6 is the only known receptor for CCL20. Western blot and immunofluorescence staining analysis revealed that co-cultured with macrophages augmented the protein expression of CCR6 in ESCs (Fig. 2C–F).

Therefore, we hypothesized that macrophages promoted the proliferation and migration of ESCs via the CCL20/CCR6 axis. To confirm this hypothesis, we treated ESCs stimulated with rCCL20 separately and added CCL20-NAb and CCR6 inhibitor, respectively, in a co-culture system to inhibit the activity of the CCL20/CCR6 axis. The results showed that the proliferation and migration capacity of ESCs stimulated with rCCL20 increased significantly as the expression of CCR6 increased (Fig. 2C–F), which was similar to the co-culture group (Fig. 2G–M). In addition, adding CCL20-NAb and CCR6 inhibitor, respectively, to the co-culture system to inhibit the activity of the CCL20/CCR6 axis counteracted the proliferation- and migration-promoting effect of macrophages on ESCs (Fig. 2G–M), as well as the increased expression of CCR6 (Fig. 2C–F).To summarize, these results indicated that macrophages activated CCR6 in ESCs by secreting CCL20, thereby promoting the proliferation and migration of ESCs.

### Macrophages block autophagic flux of ESCs through the CCL20/CCR6 axis

**Fig. 3:**
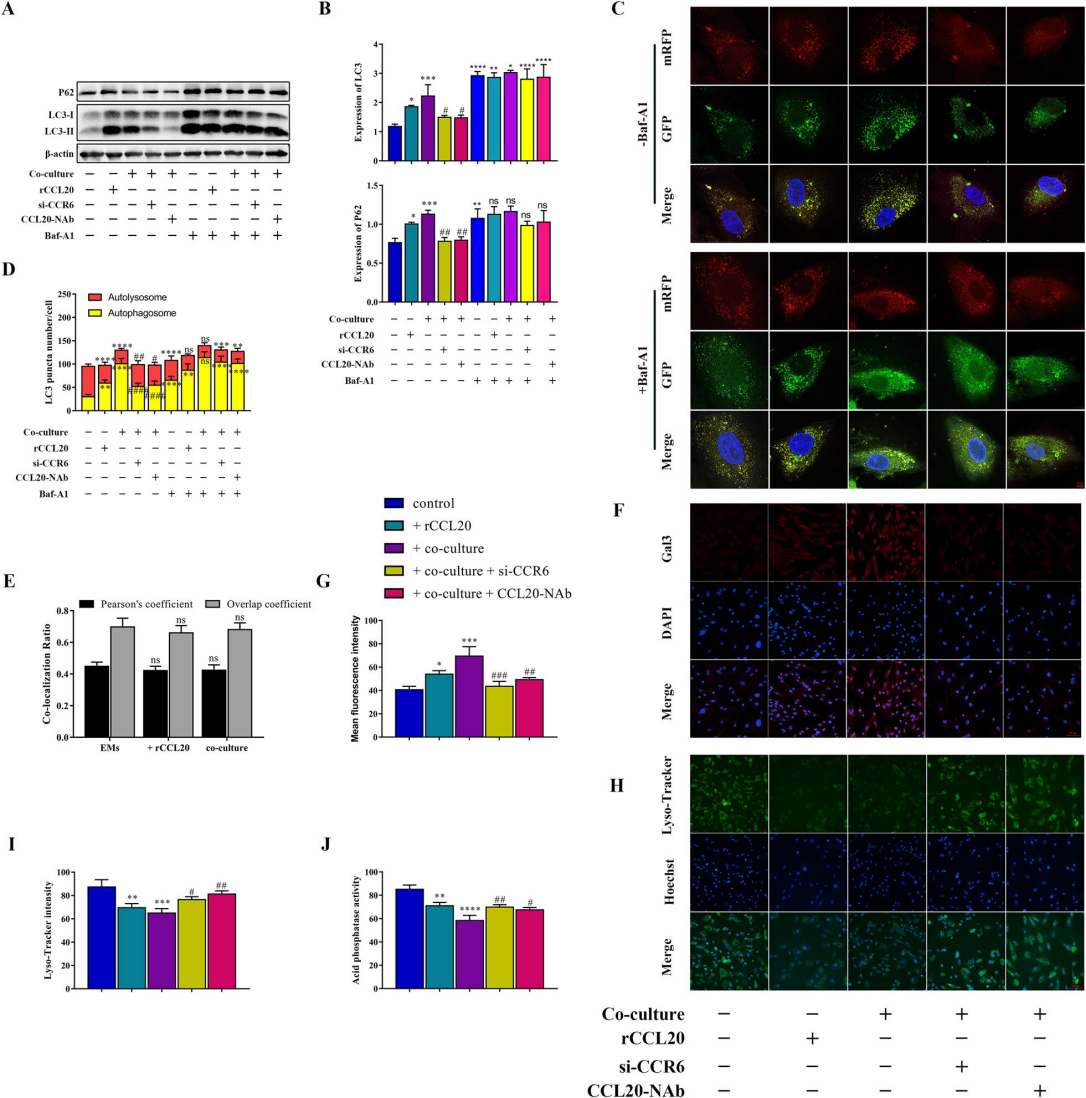
Macrophages block autophagic flux of ESCs through the CCL20/CCR6 axis. **A** The protein level of LC3 and P62 in ESCs with different treatments by western blot. **B** Quantification of the gray value of LC3 and P62 western blot bands. **C** Measurement of autophagic flux with transfecting mRFP-GFP-LC3 double fluorescence adenovirus (Ad‐LC3), which is used to distinguish autophagosomes (mRFP^+^/GFP^+^ fluorescence, yellow puncta) and autolysosomes (mRFP^+^/GFP^−^ fluorescence, red puncta). ESCs with different treatments were transfected with Ad‐LC3 in the presence or absence of 100 nM Baf-A1 treatment to suppress the fusion of autolysosomes. Confocal images of representative cells were shown. (original magnification 1000 ×) **D** Quantification of LC3 puncta number of representative cells. **E** Co-localization coefficient data show changes in co-localization levels of LC3 and LAMP2 after treatment. **F** Representative immunofluorescence images of Gal3, a specific biomarker for lysosomal membrane damage, in different treated ESCs. (original magnification 200 ×) **G** Quantitative fluorescence intensity of Gals. **H** Representative immunofluorescence images of Lyso-Tracker Green, a lysosomal probe for detecting the acidation of lysosome, in different treated ESCs. (original magnification 200 ×) **I** Quantitative fluorescence intensity of Lyso-Tracker. **J** Quantification of acid phosphatase activity in different treated ESCs. Data are presented as the mean ± SD of n = 3 independent experiments. Statistical analysis was performed using one-way ANOVA with Tukey post hoc. ****p < 0.0001, ***p < 0.001, **p < 0.01, *p < 0.05, ns, non-significant vs. control group. ^####^*p* < 0.0001, ^####^*p* < 0.001, ^##^*p* < 0.01, ^#^*p* < 0.05 vs. co-culture group. ****p < 0.0001, ***p < 0.001, **p < 0.01, *p < 0.05, ns, non-significant vs, -Baf-A1 group

In order to explore the underlying mechanism by which macrophage-mediated CCL20/CCR6 axis favors ESC proliferation and migration, we detected the protein expression of some molecules of signaling pathways related to cell proliferation and migration in ESCs by western blot, such as NF-kB, ERK1/2, JAK-STAT and autophagy pathway (Additional file 3: Fig. S3). Among these, autophagy is a pathway with obvious differences in gene expression. Many studies have indicated that autophagy-related genes are associated with the CCL20/CCR6 axis. Autophagy is a mechanism of degrading intracellular components governed by multiple steps and supports cells to adapt and respond to several processes, including proliferation, differentiation and migration. To test whether macrophages affect the autophagic flux of ESCs, we analyzed the protein expression levels of LC3 and P62 by western blot and measured the autophagic-associated structures per stromal cell transfected with the mRFP-GFP-LC3 double fluorescence adenovirus (Ad‐LC3) to evaluate autophagic flux after co-culture treatment. As shown in the merged fluorescence images, the yellow puncta represent autophagosomes (mRFP^+^/GFP^+^ fluorescence), while the red puncta represent autolysosomes (mRFP^+^/GFP^−^ fluorescence). The results showed that both LC3 and P62 protein levels were markedly augmented after co-culture treatment compared with the control group (Fig. 3A, B). The determination of mRFP-GFP-LC3 assay also revealed that co-culture treatment induced the strong accumulation of yellow puncta. These results suggested that co-cultured with macrophages blocked the later stages of autophagic flux of ESCs (Fig. 3C, D).

Additionally, we found that the expression of LC3 and P62 proteins in ESCs stimulated with rCCL20 was enhanced (Fig. 3A, B). Meanwhile, rCCL20 increased the number of yellow puncta in ESCs (Fig. 3C, D). The result was similar to that shown in the co-culture group. Additionally, adding CCL20-NAb to the co-culture system or pretreating ESCs with CCR6 knockdown (si-CCR6), respectively, in the co-culture system counteracted the elevated expression of LC3 and P62 proteins and the increased accumulation of yellow puncta in ESCs (Fig. 3A–D). These data revealed that inhibiting the activity of the CCL20/CCR6 axis could rescue the blocked autophagic flux effect of macrophages on ESCs. Together, these results demonstrated that macrophages blocked the autophagy flux of ESCs in the later stages through the CCL20/CCR6 axis.

To further determine whether macrophages blocked the fusion or degradation of autophagic flux by the CCL20/CCR6 axis in ESCs, we quantified the co-localization between LC3 and LAMP2 in ESCs and showed that the CCL20/CCR6 axis did not alter the co-localization of LC3 and LAMP2 in ESCs, suggesting that autophagosome–lysosome fusion was not blocked (Fig. 3E). As a consequence, we speculated that the degradation of autophagic substrates depending on lysosomes is blocked. To verify this speculation, we examined the lysosomal function of ESCs, including lysosomal membrane integrity and degradation capacity. Membrane-damaged lysosomes were stained with Gal3 by immunofluorescence staining. The results showed that the CCL20/CCR6 axis impaired lysosomal membrane integrity (Fig. 3F, G). In addition, both elevated lysosomal pH and decreased lysosomal acid phosphatase activity indicated the impairment of lysosomal degradation capacity (Fig. 3H–J). The above results suggested that the CCL20/CCR6 axis restricted the biological function of lysosomes in ESCs.

### CCR6 suppresses TFEB nuclear translocation to block autophagic flux by binding to TFEB

**Fig. 4:**
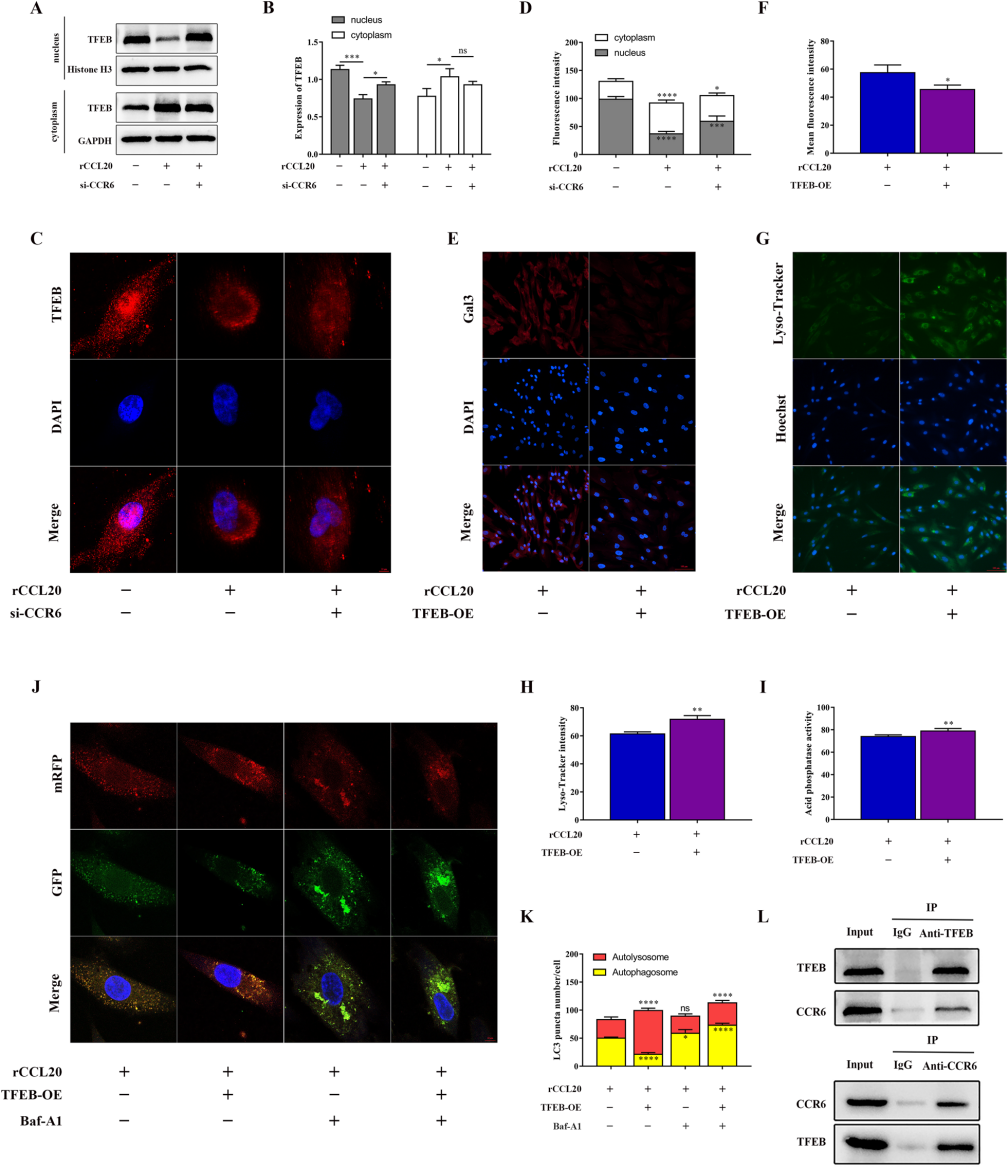
CCR6 suppresses TFEB nuclear translocation to block autophagic flux by binding to TFEB. **A** The protein level of TFEB in the nucleus and cytoplasm of ESCs with different treatments by western blot. **B** Quantification of the gray value of TFEB western blot bands in the nucleus and cytoplasm of ESCs. **C** Representative immunofluorescence images of subcellular localization of TFEB in different treated ESCs. (original magnification 1000 ×) **D** Quantitative fluorescence intensity of subcellular localization of TFEB. **E** Representative immunofluorescence images of Gal3 in different treated ESCs. (original magnification 200 ×) **F** Quantitative fluorescence intensity of Gals. **G** Representative immunofluorescence images of Lyso-Tracker Green in different treated ESCs. (original magnification 200 ×) **H** Quantitative fluorescence intensity of Lyso-Tracker. **I** Quantification of acid phosphatase activity in different treated ESCs. **J** Representative confocal images of ESCs with different treatments were transfected with Ad‐LC3 in the presence or absence of 100 nM Baf-A1 treatment. (original magnification 1000 ×) **K** Quantification of LC3 puncta number of representative cells. **L** Binding relationship between CCR6 and cytosolic TFEB by co-IP experiment. Data are presented as the mean ± SD of n = 3 independent experiments. Statistical analysis was performed using one-way ANOVA with Tukey post hoc (**B**, **D**, **K**) or Mann–Whitney test (**F**, **H**, **I**). ****p < 0.0001, ***p < 0.001, **p < 0.01, *p < 0.05, ns, non-significant

Transcription factor EB (TFEB) is known to act as a key regulator of lysosome function. To determine whether the CCL20/CCR6 axis inhibited lysosomal function by suppressing TFEB nuclear translocation (a sign of TFEB activation), we analyzed the subcellular localization of TFEB and the expression level of TFEB protein in the cytoplasm and nucleus of ESCs. Surprisingly, we observed that the CCL20/CCR6 axis inhibited the nuclear translocation of TFEB in ESCs (Fig. 4A–D). However, overexpression of TFEB eliminated the suppressive effect of the CCL20/CCR6 axis on lysosomal function (Fig. 4E–I). These results demonstrated that the CCL20/CCR6 axis inhibited lysosomal function by inhibiting TFEB nuclear translocation in ESCs. Additionally, we also verified that overexpression of TFEB can restore the effect of the CCL20/CCR6 axis blocking the autophagic flux of ESCs (Fig. 4J, K).

To explore the underlying mechanism by which the CCL20/CCR6 axis inhibits TFEB nuclear translocation, we considered whether there was an interaction between CCR6 and TFEB. Next, we confirmed the binding relationship between CCR6 and cytosolic TFEB by a co-IP assay (Fig. 4L). Taken together, these data suggested that CCR6 suppressed TFEB nuclear translocation by binding to TFEB in ESCs, which in turn impaired lysosomal function and blocked autophagic flux.

### CCL20 promotes ESC proliferation and migration via the CCR6/TFEB/Autophagy pathway

**Fig. 5:**
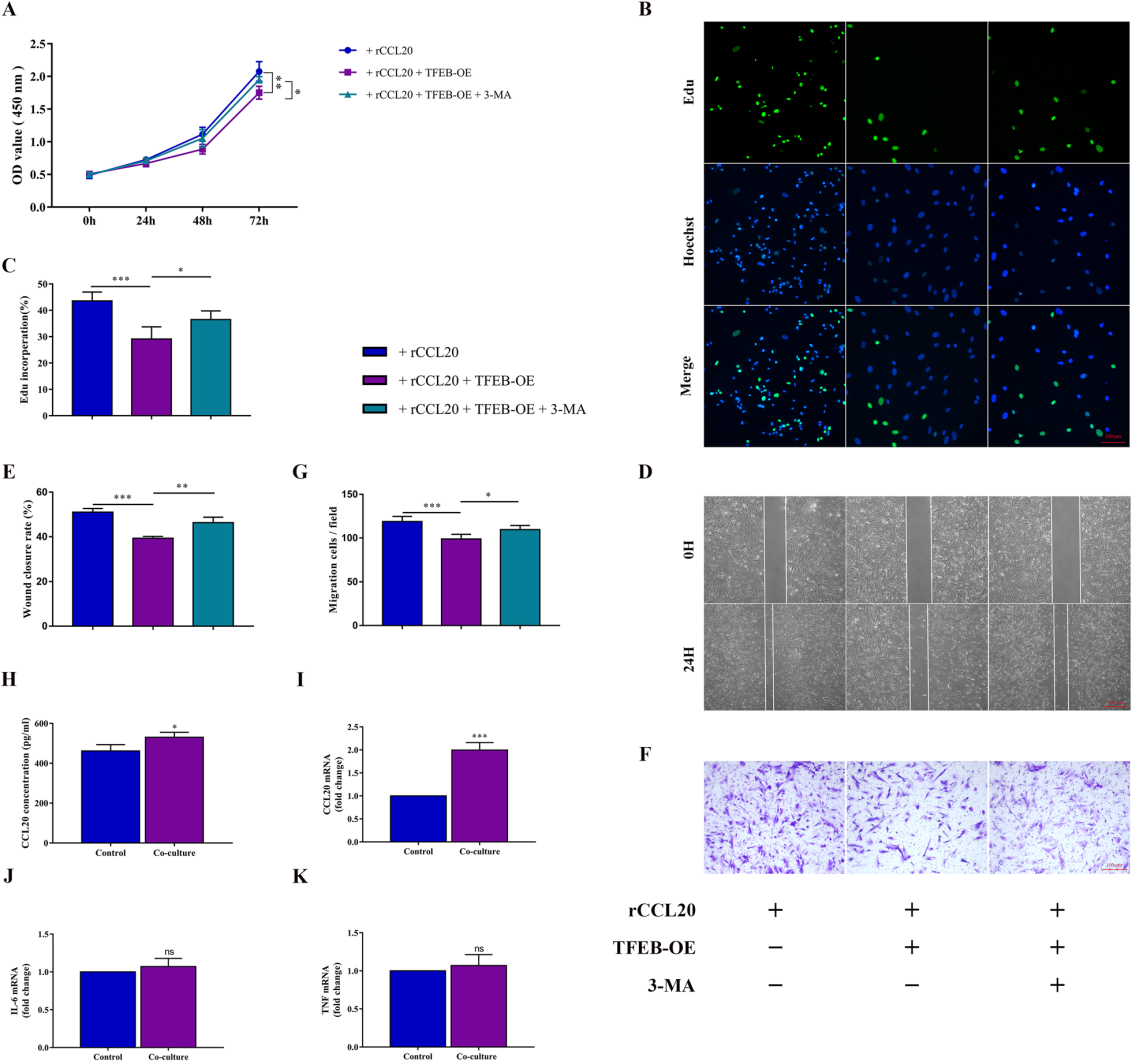
CCL20 promotes ESC proliferation and migration via the CCR6/TFEB/Autophagy pathway. **A** Proliferative capability of ESCs, incubated with rCCL20 in response to TFEB-OE with and without 3-MA (inhibitor of autophagy), assessed by CCK8 assay. **B** Proliferative capability of ESCs in different groups assessed by Edu assay. (original magnification 200 ×) **C** Quantification of Edu incorporation rate. **D** Migration capability of ESCs in different groups assessed by wound healing assay. (original magnification 40 ×) **E** Quantification of wound closure rate. **F** Migration capability of ESCs in different groups assessed by Transwell assay. (original magnification 200 ×) **G** Quantification of migration cells per field at 200 × magnification. **H** ELISA analysis of CCL20 in macrophages culture supernatant with or without co-cultured with ESCs. **I** The mRNA level of CCL20 in macrophages with or without co-cultured with ESCs by qRT-PCR. **J** The mRNA level of IL-6 in ESCs with or without co-cultured with macrophages by qRT-PCR. **K** The mRNA level of TNF-α in ESCs with or without co-cultured with macrophages by qRT-PCR. Data are presented as the mean ± SD of n = 3 independent experiments. Statistical analysis was performed using one-way ANOVA with Tukey post hoc (**A**, **C**, **E**, **G**), Mann–Whitney test (**I**, **J**, **K**) or Student’s *t* test (**H**). ***p < 0.001, **p < 0.01, *p < 0.05, ns, non-significant

We further verified whether the CCL20/CCR6 axis promoted proliferation and migration through the above blockade of the autophagy-related pathway. The results showed that overexpression of TFEB to rescue the impaired autophagic flux blocked by the CCL20/CCR6 axis could counteract the proliferation- and migration-promoting effect of the CCL20/CCR6 axis in ESCs, while restraint of autophagy flux could restore this effect (Fig. 5A–G).

What's more, we found that the production and secretion of CCL20 were upregulated by macrophages co-cultured with ESCs (Fig. 5H, I). Studies have proven that macrophages augment the secretion of CCL20 in inflammatory stimuli, such as TNF-α, IL-6 and other proinflammatory factors. Therefore, we examined the changes in the mRNA levels of ESC proinflammatory factors, including TNF-α and IL-6, after co-culture with macrophages. The data revealed that the expression of proinflammatory factors in ESCs after co-culture with macrophages increased, but did not reach statistical significance (Fig. 5J, K).

**Fig. 6:**
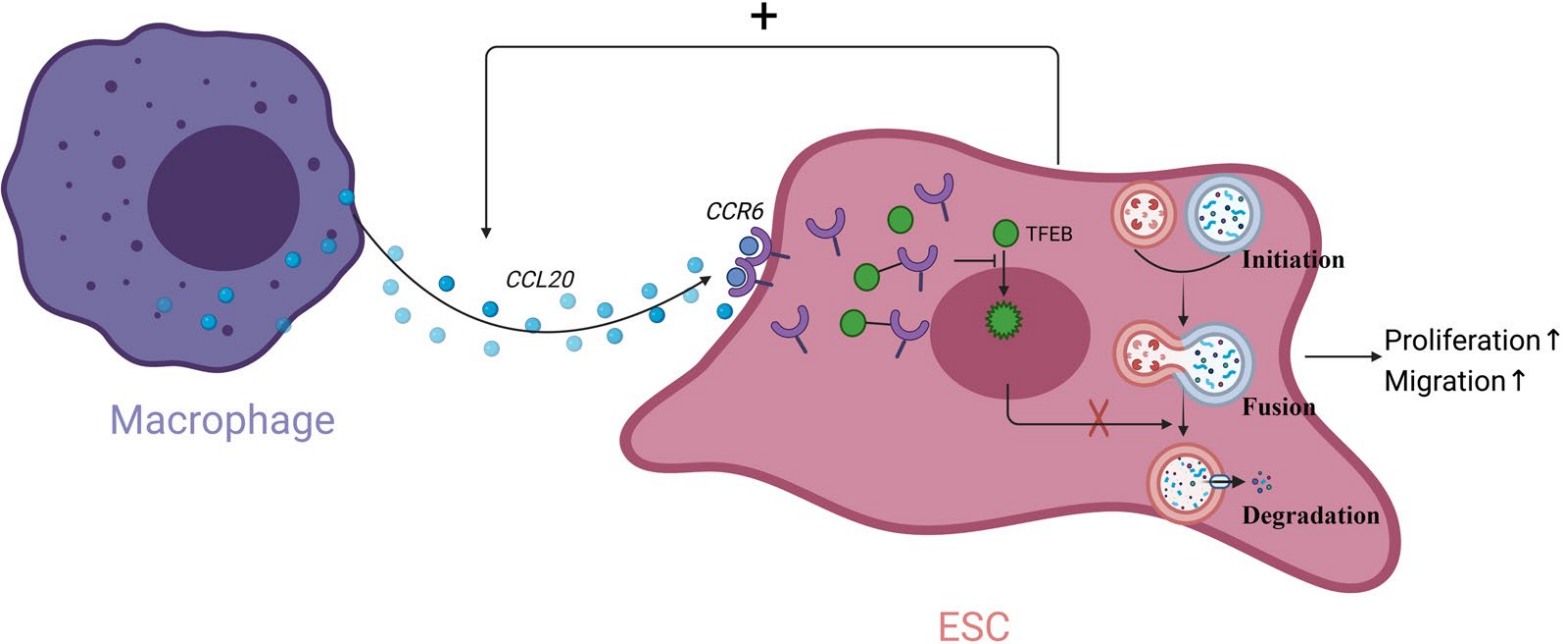
Cartoon illustration of macrophages promoting the proliferation and migration of ESCs via CCL20/CCR6/TFEB/autophagy pathway (created in BioRender.com)

Collectively, these results indicated a positive feedback loop between ESCs and macrophages, in which CCL20 secreted by macrophages promoted proliferation and migration of ESCs, while in turn enhancing the production of CCL20 from macrophages (Fig. 6).

### Blocking the CCL20/CCR6 axis suppresses the progression of EMs in vivo

**Fig. 7:**
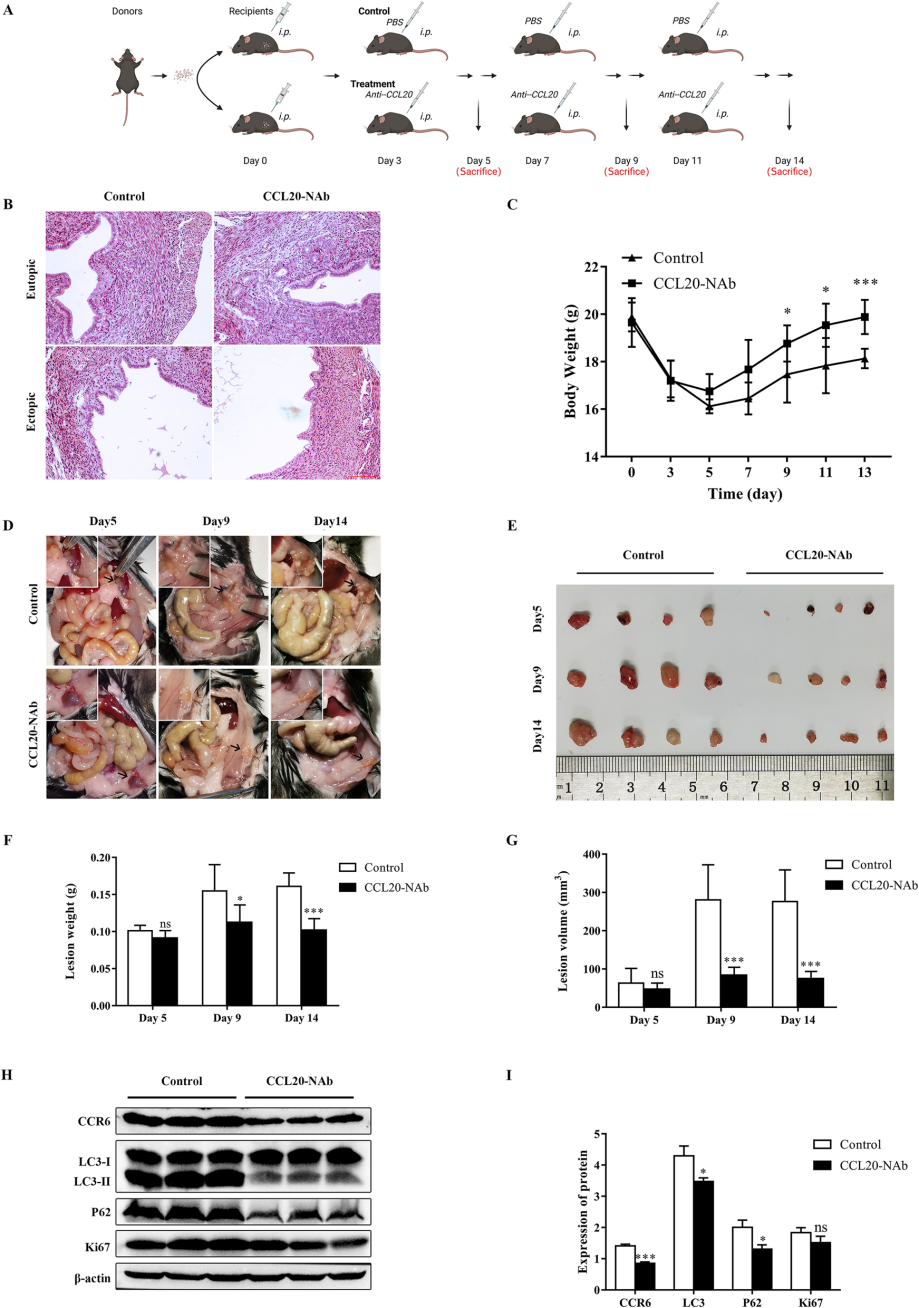
Blocking CCL20/CCR6 axis suppresses the progression EMs in vivo. **A** Cartoon illustration of animal experiments protocol (created in BioRender.com). n = 6 mice in each group per time point. **B** HE staining of mice uterus and ectopic lesions from CCL20-NAb-treated group and control group. (original magnification 200 ×) **C** Data for body weight of mice in different groups at different time points. **D** Representative images of ectopic lesions in CCL20-NAb-treated group and control group mice at day 5, day 9, day 14. Black arrowheads point to ectopic lesions. **E** Macrograph of representative ectopic lesions in two groups at day 5, day 9, day 14. **F** Data for lesion weight of two groups mice at day 5, day 9, day 14. **G** Data for lesion volume of two groups mice at day 5, day 9, day 14. **H** The protein level of CCR6, LC3, P62 and Ki67 in ectopic lesions from two groups by western blot. **I** Quantification of the gray value of CCR6, LC3, P62 and Ki67 western blot bands in ectopic lesions from two groups. Data are presented as mean ± SD. Statistical analysis was performed using Mann–Whitney test (**C**, **F**, **G**, **I**). ***p < 0.001, *p < 0.05, ns, non-significant

In order to confirm the effect of the CCL20/CCR6 axis in vivo, an EMs mice model was successfully established by intraperitoneal injection of endometrial tissue fragments. The CCL20-NAb-treated group was administered CCL20-NAb intraperitoneally every 4 days from day 3 after modeling, while the control group was injected intraperitoneally with PBS at the same time (Fig. 7A). HE staining of mice uteri and ectopic lesions from the CCL20-NAb-treated group and control group is shown (Fig. 7B). Our data indicated that CCL20-NAb treatment could ameliorate the loss in body weight caused by endometriosis lesions (Fig. 7C). Additionally, lesion weights and sizes in the CCL20-NAb-treated group were attenuated compared with those observed in the control group (Fig. 7D–G). The effect of CCL20-NAb on blocking the CCL20/CCR6 axis in ectopic lesions from EMs mice was validated by western blot (Fig. 7H, I). The data indicated that CCL20-NAb markedly decreased the protein expression of LC3 and P62 and also reduced the protein expression of Ki67 despite no statistical significance. Together, these results verified the effect of CCL20-NAb treatment on suppressing the progression of EMs in vivo.

## Discussion

In this study, we demonstrated for the first time that macrophages facilitated endometriosis progression via the CCL20/CCR6 axis. Macrophages activate CCR6 in ESCs by secreting CCL20 to block autophagic flux and then promote ESC proliferation and migration. The mechanisms involved in the above process were as follows: CCR6 binding to cytosolic TFEB to suppress TFEB nuclear translocation impaired its regulatory lysosomal function, which led to impaired degradation of autophagic flux. The in vivo suppressive effect of CCL20/CCR6 axis inhibitors on endometriosis lesions was also demonstrated in mice models.

A dysregulated immune microenvironment is a central feature of EMs, and the infiltration of macrophages, a major type of innate immune cell, has been confirmed in past studies. Our previous study demonstrated that macrophages are recruited to EMs lesions via CCL2 induced by ERβ and in turn promote the development of endometriosis. Consistently, some other studies report that macrophages contribute to the progression of EMs by enhancing cell proliferation, invasion and neoangiogenesis [46, 47]. In this study, we also demonstrated the infiltration of macrophages by immunohistochemical staining of endometriosis sections. In addition, ESCs co-cultured with macrophages exhibited an enhanced capability to proliferate and migrate compared with that in the control group. Macrophages, major secretory immune cells, are thought to produce and release a diverse repertoire of chemokines and cytokines which are crucial mediators in promoting ESC proliferation and migration. However, the chemokine and cytokine profile of macrophages in EMs remains to be adequately elucidated.

CCL20, alternatively named macrophage inflammatory protein-3α (MIP-3α), liver and activation-regulated chemokine (LARC) or Exodus-1, is the specific ligand of CCR6. The CCL20/CCR6 axis participates in the chemoattraction of immune cells. It has been reported that CCL20 expression by pleural mesothelial cells could partly facilitate the recruitment and differentiation of helper T cells in tuberculous pleurisy [48]. Hepatocellular carcinoma (HCC) cells promote the chemoattraction of CCR6^+^ B lymphocytes by secreting CCL20 and facilitate cancer development by enhancing angiogenesis [49]. IL-37 overexpression in HCC cells contributes to the recruitment and activation of dendritic cells by releasing more CCL20, participating in antitumor immunity responses [50]. In addition, CCL20 derived from macrophages contributes to the growth and invasion of several tumors and autoimmune diseases. In ovarian cancer, cisplatin-stimulated macrophages increase the production of CCL20 and activate the expression of CCR6 on cancer cells, which promotes cell migration [19]. TAMs are involved in tumor cell migration through the activated AKT signaling pathway via the CCL20/CCR6 axis in renal cell carcinoma [21]. However, the function of the CCL20/CCR6 axis in the interactions between macrophages and ESCs in EMs remains undiscovered. Here, we demonstrated the existence of CCL20 in a co-culture system of macrophages and ESCs and further explored the role of CCL20 derived from macrophages in regulating ESCs. We observed that rCCL20 contributed to ESC proliferation and migration consistent with co-culture treatment, while inhibiting the CCL20/CCR6 axis eliminated the pro-proliferation and migration effects of macrophages. These data suggested that the CCL20/CCR6 axis was required for macrophages to facilitate the proliferation and migration of ESCs in EMs.

We detected the protein expression of the molecules in some signaling pathways related to cell proliferation and migration to explore the mechanism by which macrophage-mediated CCL20/CCR6 axis facilitates ESC proliferation and migration. We then focused on the effect of the CCL20/CCR6 axis on autophagy due to obvious alterations. It has been demonstrated that the CCL20/CCR6 axis is associated with autophagy-related genes. Autophagy is a dynamic degradation process contributing to maintaining intracellular homeostasis in which damaged cellular organelles and proteins are eliminated by autolysosomes. A considerable amount of evidence supports the double-edged sword effect of autophagy, which functions as both an important early suppressor and a major advanced promotor in different stages of several tumors. Previous studies indicate that autophagy is involved in the progression of endometriosis by regulating cell proliferation, apoptosis, migration and the inflammatory response [35, 37, 51–53]. It has been reported that autophagy is induced in endometriotic cyst stromal cells treated with dienogest through repressing AKT and ERK1/2 activity which in turn impedes the activation of mTOR and facilitates apoptosis. In this study, we investigated the effect of the CCL20/CCR6 axis on autophagy in ESCs in a co-culture system with macrophages. Our data suggest for the first time that CCL20/CCR6 axis contributes to blocking autophagic flux in the degradation process of autolysosomes in ESCs co-cultured with macrophages, thereby inducing cell proliferation and migration.

Transcription factor EB (TFEB) is a key regulator of lysosomal function that is involved in the degradation process of autophagic flux [54]. Activated TFEB translocates from the cytoplasm to the nucleus and binds to the coordinated lysosomal expression and regulation element of target genes, enhancing lysosomal function [55, 56]. The role of lysosomal dysfunction mediated by inhibition of TFEB on autophagic flux has been demonstrated in numerous diseases [57–59]. In this study, we observed that the CCL20/CCR6 axis suppresses TFEB nuclear translocation, which in turn impairs lysosomal function and the degradation process of autophagic flux. Additionally, we observed that CCR6 activated by macrophage-secreted CCL20 was partly internalized into the cytosol of ESCs from the surface and bound to cytosolic TFEB which suppressed its nuclear translocation. However, whether the internalization of CCR6 results from the normal effect of macrophage-secreted CCL20 stimulation or the effect of site-specific phosphorylation and mutation on CCR6 activated by different cellular signals [60, 61] has not yet been identified and requires more effort in the future.

Another interesting finding was that macrophages produced and secreted more CCL20 when co-cultured with ESCs. Therefore, we speculated the existence of a positive feedback loop in which CCL20 was the crucial factor in the interaction between macrophages and ESCs. CCL20 is upregulated by inflammatory stimulation, such as the proinflammatory factor IL-6 and TNF-α [62–64]. Our data revealed that the expression of the proinflammatory factors TNF-α and IL-6 in ESCs after co-culture with macrophages increased, but did not reach statistical significance. Thus, the exact mechanism of how ESCs upregulate the production and secretion of CCL20 in macrophages remains to be fully elaborated in our future research. It has been demonstrated that elevated CCL20 concentrations in EMs could recruit more Treg and Th17 cells. Therefore, it is speculated that the enhanced secretion of CCL20 due to the interaction between macrophages and ESCs recruits more Tregs and TH17 cells to participate in promoting the establishment of the immunosuppressive microenvironment of EMs and protect ectopic tissues from immune system clearance. Thus, the macrophage-mediated CCL20/CCR6 axis is involved in the pathogenesis of EMs by promoting the proliferation and migration of ESCs as well as being closely related to immune dysfunction.

We validated the in vivo suppressive effect of CCL20-NAb on the CCL20/CCR6 axis in EMs mice model, and our data suggested that CCL20-NAb inhibited the growth of lesions. In addition, CCL20-NAb reduced the protein expression of LC3 and P62 in ectopic lesions. However, it was not certain that CCL20-NAb diminished the total level of autophagy in lesions or restored the blocked autophagic flux mediated by macrophages as it did in vitro experiments on ESCs, which requires more experiments to explain. Overall, since the attenuated effect of CCL20-NAb on EMs ectopic lesions was demonstrated in vivo, it can be expected that CCL20-NAb might be a candidate for clinical use in the therapy of EMs in future.

## Conclusions

In conclusion, the CCL20/CCR6 axis mediates macrophages to promote the proliferation and migration of ESCs by blocking TFEB-mediated lysosomal degradation of autophagic flux in EMs, and it might be a novel target for EMs treatment.

## Supplementary Information

**Additional file 1: Fig. S1**. Identification of primary endometriotic stromal cells (ESCs). ESCs were identified by immunofluorescent staining for vimentin (+) and cytokeratin 7 (-).

**Additional file 2: Fig. 2**. Validation of transfection efficiency. (A) Transfection efficiency of si-CCR6 assessed by qRT-PCR. (B) Transfection efficiency of TFEB-OE assessed by qRT-PCR. (C) Transfection efficiency of Ad-LC3 at different MOI visualized under fluorescence microscopy. (original magnification 200×) Data are presented as the mean ± SD of n=3 independent experiments. Statistical analysis was performed using Mann-Whitney test. ****p<0.0001, **p<0.01.

**Additional file 3: Fig. 3**. Expression of related signaling pathway genes in ESCs in response to activation of CCL20/CCR6 axis. (A) The protein level of different signaling genes in ESCs in response to activation of CCL20/CCR6 axis by western blot, including molecules of autophagy pathway (LC3, P62), ERK1/2 pathway (p-ERK1/2, ERK1/2), NF-kB pathway (NF-kB1, P65) and JAK/STAT pathway (p-JAK2, JAK2, p-STAT3, STAT3). (B) Quantification of the gray value of CCR6 western blot band. (C) Quantification of the gray value of LC3 and P62 western blot bands. (D) Statistical analysis of p-ERK1/2/ERK1/2 ratio. (E) Quantification of the gray value of P65 and NF-kB1 western blot band. (F) Statistical analysis of p-JAK2/JAK2 ratio and p-STAT3/STAT3 ratio. Data are presented as the mean ± SD of n=3 independent experiments. Statistical analysis was performed using Mann-Whitney test. **p<0.01,*p<0.05, ns, nonsignificant.

## Data Availability

The datasets used and/or analyzed during the current study are available from the corresponding author on reasonable request.

## Abbreviations

Ad-LC3: MRFP-GFP-LC3 double fluorescence adenovirus
CCL20: CC chemokine ligand 20
CCL20-NAb: CCL20 neutralizing antibody
CCR6: Chemokine (C–C motif) receptor 6
co-IP: Co-immunoprecipitation
DMEM: Dulbecco’s modified Eagle medium
ELISA: Enzyme-linked immunosorbent assay
EMs: Endometriosis
EMT: Epithelial–mesenchymal transition
ESC: Endometriotic stromal cells
FBS: Fetal bovine serum
HCC: Hepatocellular carcinoma
LARC: Liver and activation-regulated chemokine
MIP-3α: Macrophage inflammatory protein-3α
PMA: Phorbol 12-myristate 13-acetate
qRT-PCR: Quantitative real-time PCR (qRT-PCR)
rCCL20: Recombinant human CCL20
RPMI-1640: Roswell Park Memorial Institute 1640
TAMs: Tumor-associated macrophages
TFEB: Transcription factor EB; 3-MA: 3-Methyladenine
