# Supplementary figures and images for: CCL20/CCR6 axis mediates macrophages to promote proliferation and migration of ESCs by blocking autophagic flux in endometriosis
Source: Stem Cell Res Ther. 2022 Jul 15;13:294. doi: 10.1186/s13287-022-02981-2 (PMC9284876; doi:10.1186/s13287-022-02981-2)

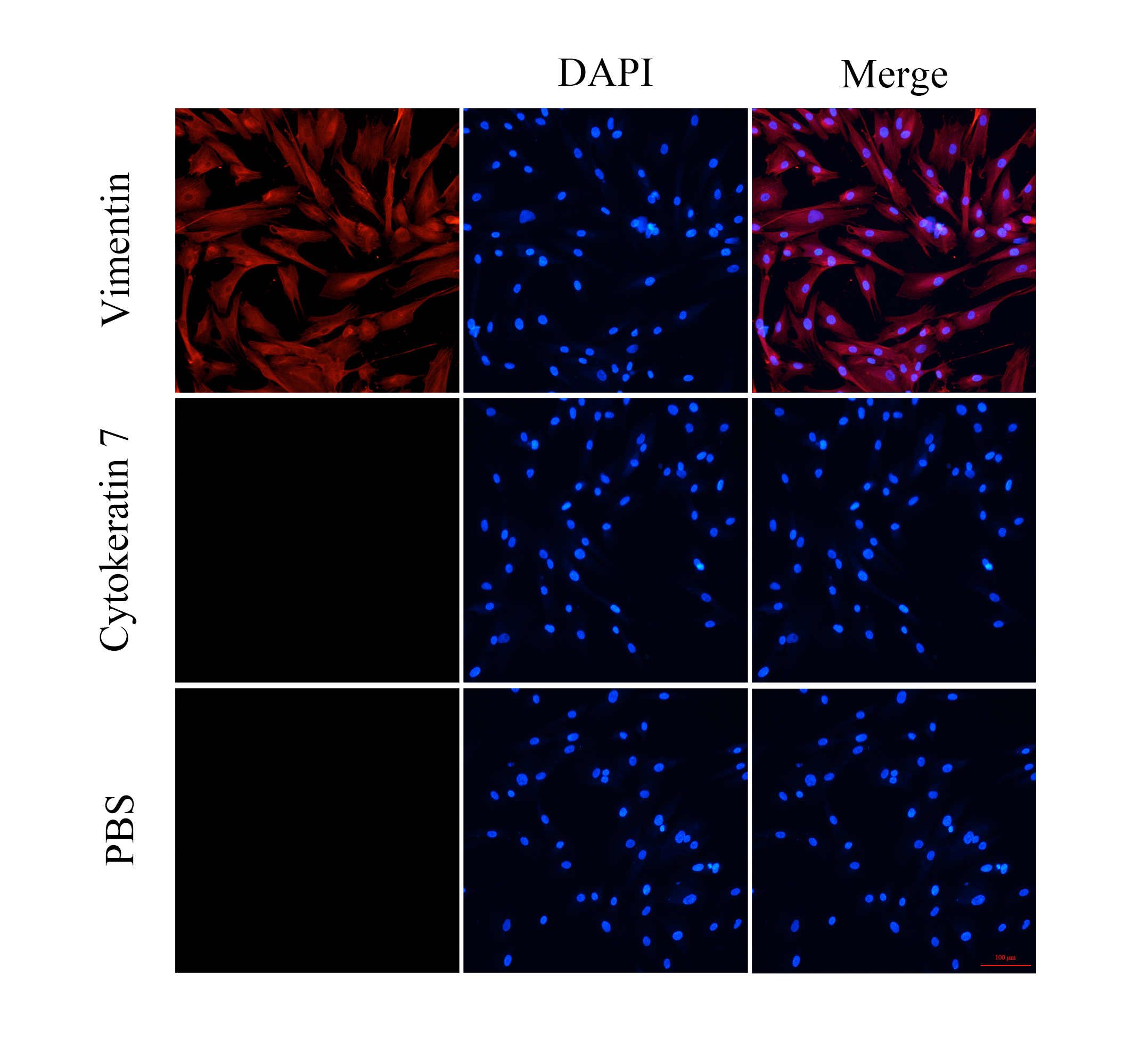

Supplement: Supplementary file 1 — Additional file 1: Fig. S1. Identification of primary endometriotic stromal cells (ESCs). ESCs were identified by immunofluorescent staining for vimentin (+) and cytokeratin 7 (-). [file 13287_2022_2981_MOESM1_ESM.tif]

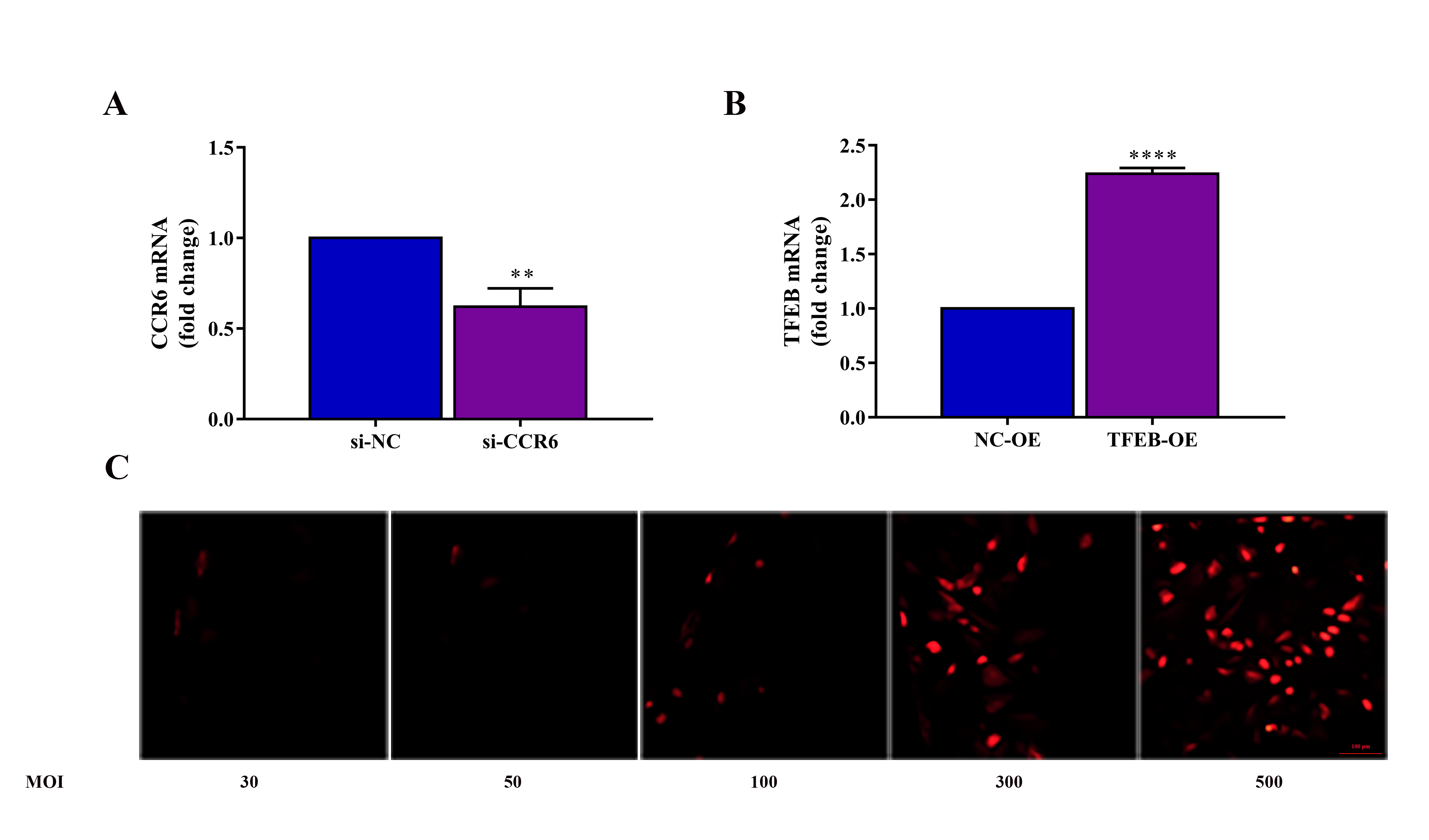

Supplement: Supplementary file 2 — Additional file 2: Fig. 2. Validation of transfection efficiency. (A) Transfection efficiency of si-CCR6 assessed by qRT-PCR. (B) Transfection efficiency of TFEB-OE assessed by qRT-PCR. (C) Transfection efficiency of Ad-LC3 at different MOI visualized under fluorescence microscopy. (original magnification 200×) Data are presented as the mean ± SD of n=3 independent experiments. Statistical analysis was performed using Mann-Whitney test. ****p<0.0001, **p<0.01. [file 13287_2022_2981_MOESM2_ESM.tif]

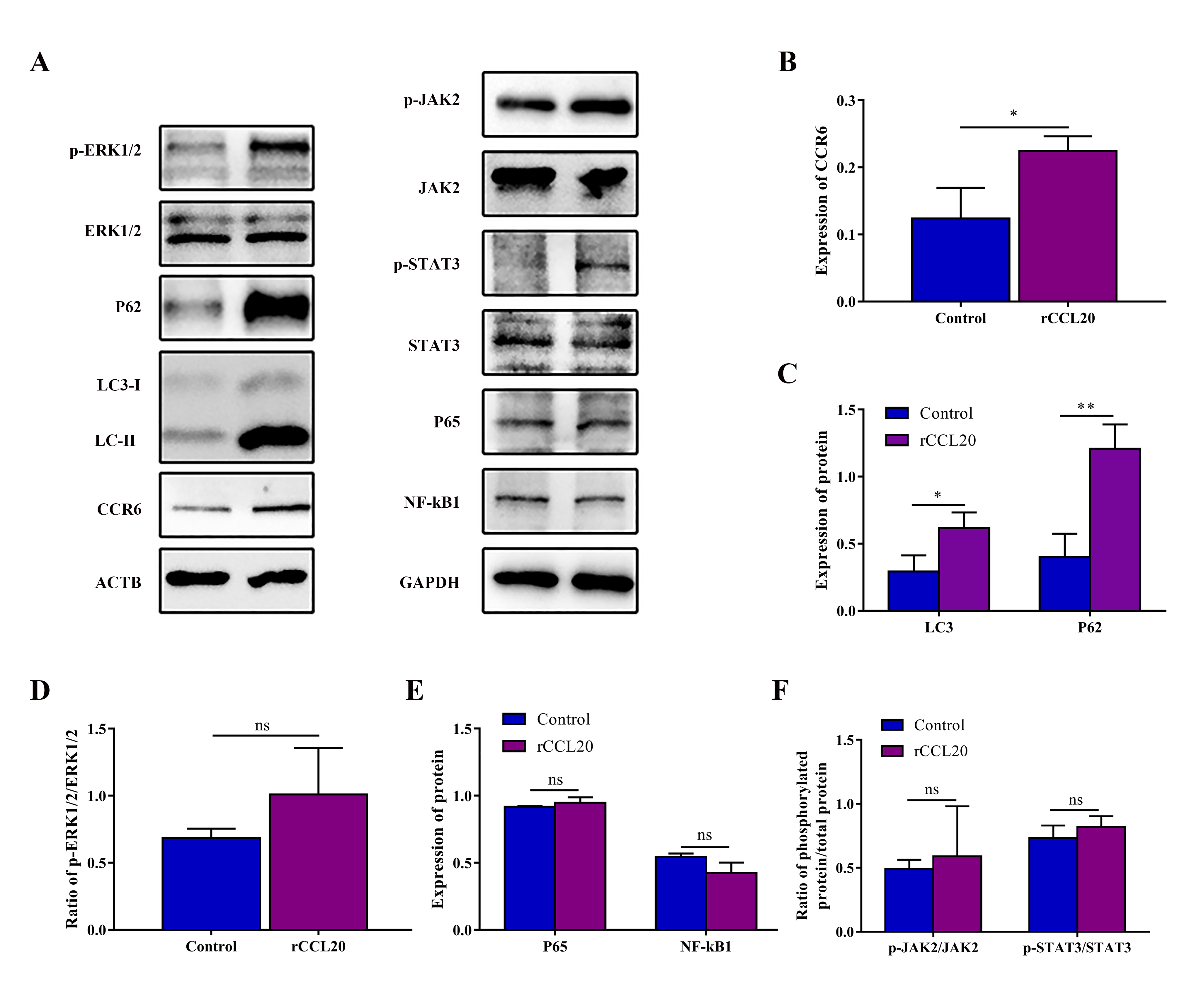

Supplement: Supplementary file 3 — Additional file 3: Fig. 3. Expression of related signaling pathway genes in ESCs in response to activation of CCL20/CCR6 axis. (A) The protein level of different signaling genes in ESCs in response to activation of CCL20/CCR6 axis by western blot, including molecules of autophagy pathway (LC3, P62), ERK1/2 pathway (p-ERK1/2, ERK1/2), NF-kB pathway (NF-kB1, P65) and JAK/STAT pathway (p-JAK2, JAK2, p-STAT3, STAT3). (B) Quantification of the gray value of CCR6 western blot band. (C) Quantification of the gray value of LC3 and P62 western blot bands. (D) Statistical analysis of p-ERK1/2/ERK1/2 ratio. (E) Quantification of the gray value of P65 and NF-kB1 western blot band. (F) Statistical analysis of p-JAK2/JAK2 ratio and p-STAT3/STAT3 ratio. Data are presented as the mean ± SD of n=3 independent experiments. Statistical analysis was performed using Mann-Whitney test. **p<0.01,*p<0.05, ns, nonsignificant. [file 13287_2022_2981_MOESM3_ESM.tif]
